# Indocyanine green and poly I:C containing thermo-responsive liposomes used in immune-photothermal therapy prevent cancer growth and metastasis

**DOI:** 10.1186/s40425-019-0702-1

**Published:** 2019-08-14

**Authors:** Li Xu, Wei Zhang, Hae-Bin Park, Minseok Kwak, Junghwan Oh, Peter C. W. Lee, Jun-O Jin

**Affiliations:** 1 Shanghai Public Health Clinical Center & Institutes of Biomedical Sciences, Shanghai Medical College, Fudan University, Shanghai, 201508 China; 2 Department of Medical Biotechnology, Yeungnam University, Gyeongsan, 38541 South Korea; 3 Department of Chemistry, Pukyong National University, Busan, 48513 South Korea; 4 Marine-Integrated Bionics Research Center, Pukyong National University, Busan, 48513 South Korea; 5 Department of Biomedical Engineering and Center for Marine-Integrated Biomedical Technology (BK21 Plus), Pukyong National University, Busan, 48513 South Korea; 6 Interdisciplinary Program of Biomedical Mechanical & Electrical Engineering, Pukyong National University, Busan, 48513 South Korea; 7 Department of Biomedical Sciences, University of Ulsan College of Medicine, ASAN Medical Center, Seoul, South Korea

**Keywords:** Liposome, Indocyanine green, Polyinosinic:polycytidylic acid, Photothermal therapy, Immunotherapy, Anti-metastasis

## Abstract

**Background:**

Efficient cancer therapy is sought not only for primary tumor treatment but also for the prevention of metastatic cancer growth. Immunotherapy has been shown to prevent cancer metastasis by inducing antigen-specific immune responses. Indocyanine green (ICG) has a peak spectral absorption at about 800 nm, which makes it a photothermal reagent for direct treatment of solid tumors by photothermal therapy (PTT). Since PTT alone cannot fully induce antigen-specific immune response for prevention of cancer metastasis, the combination of PTT and immunotherapy has been developed as a new strategy of cancer treatment.

**Methods:**

Thermal responsive liposomes (TRL) were synthesized by incorporating ICG into the lipid bilayer and encapsulating the water-soluble immune stimulatory molecule polyinosinic:polycytidylic acid (poly I:C) in the hydrophilic core. The poly I:C- and ICG-containing TRLs (piTRLs) were analyzed according to size, and their photothermal effect was evaluated following laser irradiation at 808 nm. Moreover, the temperature-dependent release of poly I:C was also measured. For cancer therapy, CT-26 (carcinoma) and B16 (melanoma) cells were subcutaneously inoculated to build the 1st transplanted tumor in BALB/c and C57BL/6 mice, respectively. These mice received a 2nd transplantation with the same cancer cells by intravenous inoculation, for evaluation of the anti-metastatic effects of the liposomes after PTT.

**Results:**

Near-infrared (NIR) laser irradiation increased the temperature of piTRLs and effectively released poly I:C from the liposomes. The increased temperature induced a photothermal effect, which promoted cancer cell apoptosis and dissolution of the 1st transplanted tumor. Moreover, the released poly I:C from the piTRL induced activation of dendritic cells (DCs) in tumor draining lymph node (tdLN). Cancer cell apoptosis and DC-activation-mediated cancer antigen-specific immune responses further prevented growth of lung metastatic cancer developed following intravenous transplantation of cancer cells.

**Conclusion:**

These results demonstrated the potential usage of a piTRL with laser irradiation for immuno-photothermal therapy against various types of cancer and their metastases.

**Electronic supplementary material:**

The online version of this article (10.1186/s40425-019-0702-1) contains supplementary material, which is available to authorized users.

## Background

Photothermal therapy (PTT) has been developed as an alternative treatment strategy for tumors. This technique that uses heat-generated thermal energy to kill tumor cells by nanoparticles absorbing near-infrared (NIR) light [1–4]. PTT promotes apoptosis of cancer cells via a thermal reaction [5, 6] which is cleared by immune cells [7–9]. Indocyanine green (ICG) is a photothermal reagent used in medical diagnostics and photothermal therapy [10, 11]. ICG has a peak spectral absorption at about 800 nm, and its temperature increases upon irradiation with NIR light [10, 11]. ICG has been approved as a NIR clinical imaging agent by the Food and Drug Administration (FDA) in the USA due to low incidence rates of adverse reactions [12, 13].

Since the success of immunotherapy relies on the patient’s own immunity, the interest in this method of treatment of cancer has greatly increased [14]. Therapies such as monoclonal antibodies (Abs), immune cell transfer, immune checkpoint-inhibitors, and cancer vaccines have been developed and applied to the treatment of cancer [15–19]. Moreover, recent therapeutic trials have achieved effective treatments of cancer, which, however, have exhibited undesirable side effects such as inflammation [20–22]. In addition, the induction of antigen- (Ag-) specific immune responses is another therapeutic approach and prevention strategy against cancer. However, additional studies are required due to the lack of suitable candidates and the poor immune stimulatory effect of cancer Ags. Despite these immunotherapies, metastasis, which causes the majority of cancer-related deaths, is another obstacle faced by scientists in their efforts to cure cancer [23]. Therefore, to achieve the ultimate cancer therapy, not only must the primary cancer should be treated, but metastasis must also be prevented.

To enhance the efficiency of cancer therapeutics, researchers are studying a combination of therapies, since such an approach has been shown to have beneficial effects including the prevention of metastatic cancer and the reduction of side effects [20–22]. In this study, we developed a poly I:C and ICG containing temperature-sensitive liposomes (piTRLs). We hypothesized that piTRLs could treat primary tumors through the administration PTT and prevent metastatic lung cancer via immunotherapy in mice in vivo*;* the current study was undertaken to test this hypothesis.

## Material and methods

### Synthesis of the temperature-sensitive liposome

Liposomes (DPPC, MPPC, and DSPE-PEG2000 in the molar ratio of 86:10:4) were prepared by a thin film hydration method as described in a previous study [24]. Briefly, lipids were resuspended with chloroform, and ICG was blended in methanol (ICG: lipid = 20:1 in weight ratio). The resultant solution was removed under nitrogen gas at room temperature (RT) for 1.5 h, followed by vacuum drying for at least 4 h. The dried lipid films were hydrated at 65 °C with PBS or 1 mg/ml poly I:C solution in PBS for 1 h. Then the suspension was extruded through a polycarbonate membrane of 200 nm using a mini-extruder (Avanti Polar Lipids, Alabaster, AL).

### Determination of poly I:C concentration in liposome

The loaded concentration of poly I:C in the liposomes was determined by: isolating the fresh liposomes from the aqueous suspension medium by ultracentrifuge (20,000 rpm, 4 °C for 30 min) (Optima L-100XP, Beckman, USA). The concentration of un-encapsulated poly I:C in the buffer was measured by a GeneJET RNA Cleanup and Concentration Micro Kit (Thermo fisher scientific, Waltham, MA, USA) according to the manufacturer’s instructions. The concentration of poly I:C in the liposomes was calculated by the difference between total quantity and supernatant concentration of poly I:C after extrusion. The encapsulated efficiency of poly I:C in the liposomes was 18.7%, which was 200 μg/ml of poly I:C.

### Characterization of liposomes

Field emission transmission electron microscopy (FE-TEM) and electron diffraction (ED) pattern images were taken using a JEM-2100F transmission electron microscope (JEOL; Tokyo, Japan). UV-vis absorption spectra were recorded using a UV-visible spectrophotometer (Beckman Coulter; Fullerton, CA, USA). A fiber-coupled continuous-wave diode laser (808 nm, 10 W) was purchased from Changchun New Industries Optoelectronics Technology Co., Ltd. (Changchun, China). Thermographic images and changes of temperature were taken by a FLIR ONE (FLIR Systems, Wilsonville, OR, USA).

### Mice and cell lines

C57BL/6 mice and BALB/c mice were obtained from Shanghai Public Health Clinical Center and kept under pathogen-free conditions. The mice were maintained in a room with controlled temperature (20–22 °C), humidity (50–60%), and light (12 h: 12 h) with free access to standard rodent chow and water. Mice were euthanized by CO_2_ inhalation, and all efforts were made to minimize suffering. The murine melanoma cell line B16F10 (ATCC, CRL-6475) and the murine carcinoma cell line CT-26 (ATCC, CRL-2638) were cultured in RPMI 1640 (Sigma Aldrich, St. Louis, MO, USA) supplemented with 10% FBS, 2 mM glutamine, 1 M HEPES, 100 μg/ml streptomycin, 100 U/ml penicillin, and 2 mM 2-mercaptoethanol. All cell lines were cultured at 37 °C in a humidified atmosphere of 5% CO_2_ and air.

### Antibodies (abs)

Mouse Abs and isotype control Abs (IgG1, IgG2a or IgG2b), CD11c (HL3), CD4 (GK1.5), CD8α (YTS169.4), CD40 (3/23), CD80 (16-10A1), and CD86 (GL-1) were obtained from BioLegend (Snd Diego, CA, USA); anti-MHC class I (AF6–88.5.3) and anti-MHC class II (M5/114.15.2) Abs were obtained from eBioscience (San Diego, CA, USA).

### Flow cytometry analysis

Cells were washed with PBS containing 0.5% BSA, pre-incubated for 15 min with unlabeled isotype control Abs and Fc block Abs (BioLegend, San Diego, CA, USA), and then labeled with fluorescence-conjugated Abs by incubation on ice for 30 min followed by washing with PBS. Cells were analyzed with FACS Fortessa (Becton Dickinson, Franklin Lakes, New Jersey, USA) and FlowJo 8.6 software (Tree Star, San Diego, CA, USA). Cellular debris were excluded from the analysis by forward- and side-scatter gating. Dead cells were further excluded by 4′,6-diamidino-2-phenylindole (DAPI) (Sigma-Aldrich) staining and gating on the DAPI-negative population. As a control for nonspecific staining, isotype-matched irrelevant mAbs were used.

### In vitro photothermal treatment

CT-26 cells (1 × 10^5^) were seeded into a 24-well plate for 24 h. After 1 h of treatment, the cells were irradiated with an 808-nm laser at 1 W/cm^2^ for 5 min.

### MTT assay

CT-26 cells (2 × 10^4^) were seeded into a 96 well plate for 24 h. Then, 100 μL of freshly prepared MTT solution (5 mg/mL in PBS) was added to each well, after which 100 μL of Dimethyl sulfoxide (DMSO, Gibco; Paisley, UK) was added and incubation was initiated for an additional 4 h. The wells were analyzed by an ELISA reader at 620 nm (Labsystems Multiskan; Roden, Netherlands).

### Apoptosis assay

Cells were stained with annexin V-FITC and 7AAD in 100 μL of binding buffer for 15 min at RT. The cells were analyzed by flow cytometry using a FACS Fortessa (Becton Dickinson, Franklin Lakes, New Jersey, USA) after 400 μL of binding buffer was added without washing.

### Western blot analysis

CT-26 cells were treated with lysis buffer containing 1% Triton X-100, 10% glycerol, 137 mM NaCl, 1.5 mM MgCl2, 1 mM EGTA, and protease inhibitors. Proteins in the cell lysate were separated by 10% SDS–PAGE and transferred to nitrocellulose membranes. The membranes were incubated with a blocking buffer (10 mM Tris–HCl, 0.15 M NaCl, 0.1% NaN_3_, and 5% skim milk) for 1 h and stained with anti-procaspase-3, − 8 and − 9 Abs overnight at 4 °C. The membranes were stained with the secondary Abs for 2 h, and signals were detected using ECL chemiluminescence following the manufacturer’s instructions.

### Mouse DC analysis

Tumor-draining lymph node (tdLN) DCs were analyzed as described in other studies [25, 26]. Briefly, the tdLN were homogenized and digested with collagenase for 20 min at room temperature (RT). Cells were centrifuged into a pellet and resuspended in 5 mL of histopaque-1.077 (Sigma-Aldrich, St. Louis, MO, USA). Additional histopaque-1.077 was layered below, and 1 ml of FBS was layered above the cell suspension. The tube was centrifuged at 1700 x g for 10 min without break. The light density fraction (< 1.077 g/cm^3^) was harvested and stained with the following FITC-conjugated monoclonal Abs (mAbs) for 30 min: anti-CD3 (17A2), anti-Thy1.1 (OX-7), anti-B220 (RA3-6B2), anti-Gr1 (RB68C5), anti-CD49b (DX5), and anti-TER-119 (TER-119). The lineage^−^CD11c^+^ cells were defined as DCs, which were further divided into CD8α^+^ and CD8α^−^ DCs. Analysis was carried out on a FACS Fortessa (Becton Dickinson, Franklin Lakes, NJ, USA).

### Real-time PCR

Total RNA was reverse-transcribed into cDNA using Oligo (dT) and M-MLV reverse transcriptase (Promega, Madison, Wisconsin, US). The cDNA was subjected to real-time PCR amplification (Qiagen, Hilden, Germany) for 40 cycles with annealing and extension temperature at 60 °C on a LightCycler 480 Real-Time PCR System (Roche, Basel, Switzerland). Primer sequences were: mouse β-actin forward, 5′-TGGATGACGATATCGCTGCG-3′; reverse, 5′-AGGGTCAGGATACCTCTCTT-3′, IL-6 forward, 5′-AACGATGATGCACTTGCAGA-3′; reverse, 5′-GAGCATTGGAAATTGGGGTA-3′, IL-12p40 forward, 5′-CACATCTGCTGCTCCACAAG-3′; reverse, 5′- CCGTCCGGAGTAATTTGGTG-3′, TNF-α forward, 5′-CCTTTCACTCACTGGCCCAA-3′; reverse, 5′-AGTGCCTCTTCTGCCAGTTC-3′ T-bet forward, 5′-CAACAACCCCTTTGCCAAAG-3′; reverse, 5′-TCCCCCAAGCATTGACAGT-3′, GATA3 forward, 5′-CGGGTTCGGATGTAAGTCGAGG-3′; reverse, 5′- GATGTCCCTGCTCTCCTTGCTG-3′, RORγt forward, 5′-CCGCTGAGAGGGCTTCAC-3′; reverse 5′-TGCAGGAGTAGGCCACATTACA-3′, IFN-γ forward, 5′-GGATGCATTCATGAGTATTGC-3′; reverse, 5′-CTTTTCCGCTTCCTGAGG-3′, IL-4 forward, 5′-ACAGGAGAAGGGACGCCAT-3′; reverse 5′-GAAGCCCTACAGACGAGCTCA-3′, IL-17A forward, 5′-GCGCAAAAGTGAGCTCCAGA-3′; reverse 5′-ACAGAGGGATATCTATCAGGG-3′.

### In vivo photothermal treatment

Once tumors at their longest dimension reached a size of approximately 5.0 mm on day 7, mice were randomized into eight treatment groups: PBS, TRL, iTRL, and piTRL with or without laser irradiation. Each of the liposomes was intratumorally (*i.t.*) injected into the mice. One hour after injection an 808 nm NIR laser was applied to irradiate tumors under a power intensity of 1 W/cm^2^ for 5 min. The temperature was recorded using an infrared camera FLR One Thermal imaging system (FLIR, Wilsonwille, OR, USA). Tumor volume was calculated by using the formula V ¼ 1/2 (L/S2), where L is the longest dimension and S is the shortest dimension.

### 2nd transplanted model

BALB/c and C57BL/6 mice were intravenously (*i.v.*) injected with CT-26 and B16 cells, respectively. The survival of mice was monitored for 21 days after cancer cell injection.

### Hematoxylin and eosin staining

As described in detail in a previous study [27], colon, kidney, and liver samples were fixed in 4% paraformaldehyde, embedded in paraffin, and sectioned to 6 μm thickness. Sections were then stained with hematoxylin and eosin (H&E) and examined for inflammation and tissue damage.

### ELISPOT assay

ELISPOTs for mouse IFN-γ were performed according to the manufacturer’s protocol (Biolegend, San Diego, CA, USA). In short, IFN-γ capture Abs were pre-coated on the plate and splenocytes were seeded at 50 × 10^3^ cells/well. Fresh 2 × 10^6^ CT-26 or B16 cells were lysed by freeze and thaw, respectively. After centrifugation, suspended cancer Ag proteins were harvested, and 10 μg/mL proteins were incubated with splenocytes at 37 °C for 24 h. ELISPOT plates were counted automatically using a CTL ELISPOT reader (CTL Europe GmbH, Bonn, Germany).

### Antigen-specific lysis of splenocytes

A mixture of splenocytes labeled with CFSE (200 nM) and loaded with 1 μg/mL cancer Ag proteins, and spleen cells labeled with 10 mM CellTracker™ Orange CMTMR (Life technologies) and not loaded with protein was transferred into C57BL/6 mice. Six hours after transfer, the spleen was harvested and the population of splenocytes were analyzed by Novocyte flow cytometer and NovoExpress® software from ACEA Biosciences Inc. (San Diego, CA, USA).

### T cell depletion and blocking of co-stimulator

Anti-CD4 (GK1.5), anti-CD8 (YTS169.4), anti-CD80 (1G10) and anti-CD86 (GL-1) Abs were intraperitoneally (*i.p.*) administered in the mice on day 25 after the 1st subcutaneous inoculation of cancer cells (3 days before the 2nd intravenous administration of cancer cells). The Abs were purchased from BioXcells (West Lebanon, NH, USA) and 100 μl of 1 mg/ml Abs was administered every 3 days in the mice. The depletion of cells was confirmed using Novocyte flow cytometer (San Diego, CA, USA).

### Statistical analysis

Results are expressed as the mean ± standard error of the mean (SEM). Data sets were analyzed by one-way ANOVA using the Tukey multiple comparison test with GraphPad Prism 4. *P* values smaller than 0.05 were considered to be statistically significant.

## Results

### piTRL induced elevated temperatures and released poly I:C in response to near-infrared (NIR) light

**Fig. 1:**
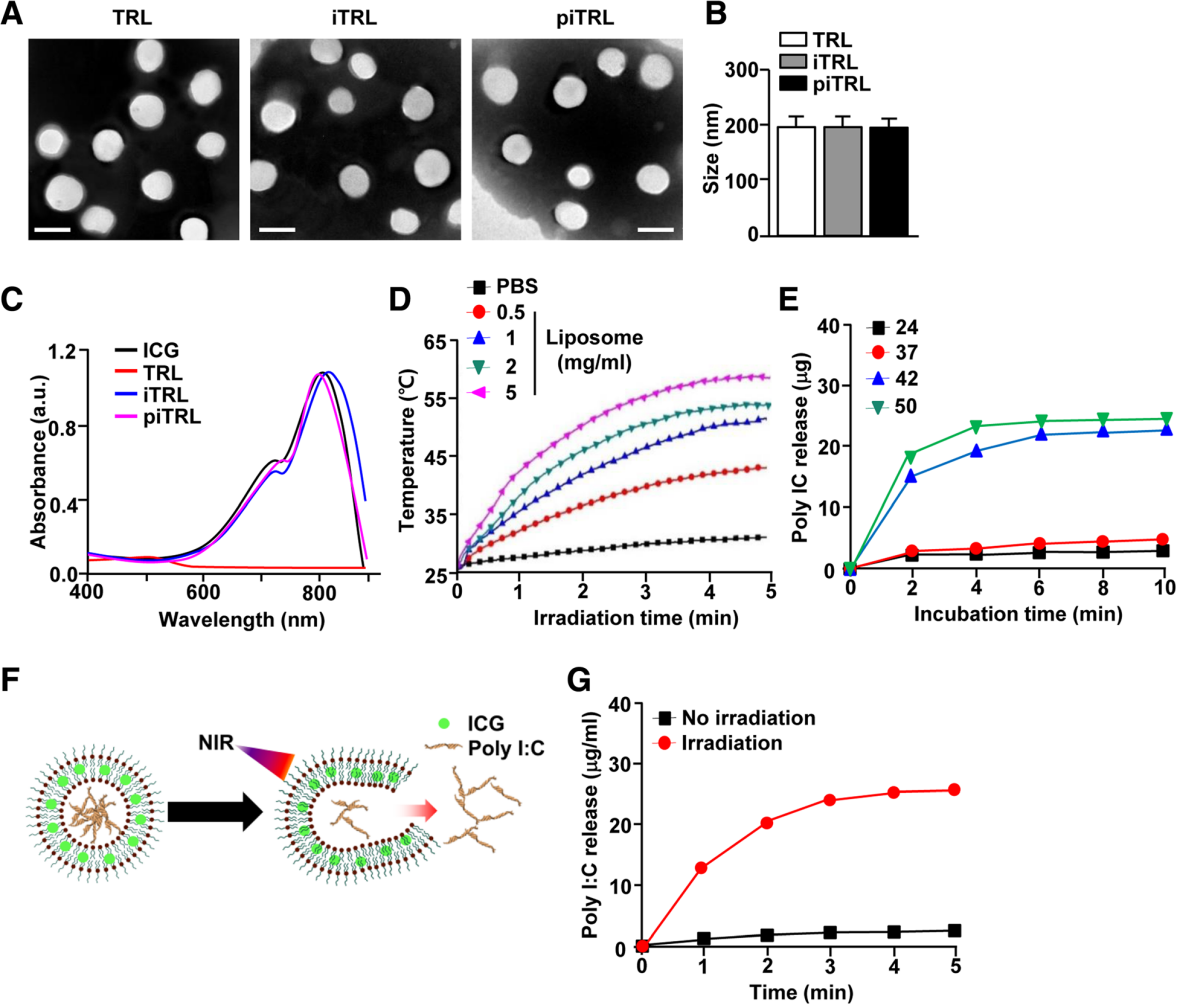
Characterization of liposomes. **a** FE-TEM images of TRL, iTRL, and piTRL. (Scale bars: 200 nm). **b** TEM corresponding size distribution of each liposome. **c** UV-vis absorption of liposomes is shown. **d** Photothermal heating curves of different concentrations of piTRL, irradiated for 5 min with an 808-nm laser at a power density of 1 W/cm^2^. **e** The cumulative release of poly I:C from piTRL at 24, 37, 42, and 50 °C. **f** Schematic diagram of poly I:C release from piTRL under NIR-laser irradiation. **g** The concentration of released poly I:C from piTRL under NIR-laser irradiation at a power intensity of 1 W/cm^2^

Using the thin-film rehydration method, TRLs (DPPC, MPPC, and DSPE-PEG2000 in a molar ratio of 86:10:4) with incorporated ICG in the lipid bilayer (iTRL) were prepared. The soluble poly I:C was then encapsulated by extrusion through a 200 nm size of polycarbonate membrane using a mini-extruder (piTRL). As shown in Fig. 1a, the liposomes were successfully synthesized, and all liposomes had similar size (Fig. 1b). Since the liposomes had incorporated ICG, we measured the absorption spectra of the liposomes. Both iTRLs and piTRLs have strong peak absorption rates at 811 and 791 nm, respectively (Fig. 1c). For evaluation of the photothermal efficiency of piTRLs, we measured the changes in temperature under laser irradiation (1 W/cm^2^) at 808 nm for 5 min and found that the piTRLs showed greater temperature increases in a dose-dependent manner when compared to phosphate-buffered saline (PBS). The temperatures of the 2 and 5 mg/mL piTRLs reached up to 50 °C and 58 °C, respectively, within 5 min, whereas the temperature of PBS increased up to 28 °C (Fig. 1d).

Since TRL is sensitive to high temperatures, we evaluated the release of poly I:C at different temperatures. Incubation of piTRLs at 24, 37, 42 and 50 °C for 5 min resulted substantial release of poly I:C from the liposomes at 42 and 50 °C (Fig. 1e). Moreover, laser irradiation also induced efficient release of poly I:C in the piTRLs within 5 min (Fig. 1f and g). Thus, these results indicated that piTRLs release poly I:C and produce a photothermal effect.

### piTRL and laser irradiation induced apoptosis of cancer cell by photothermal effect

**Fig. 2:**
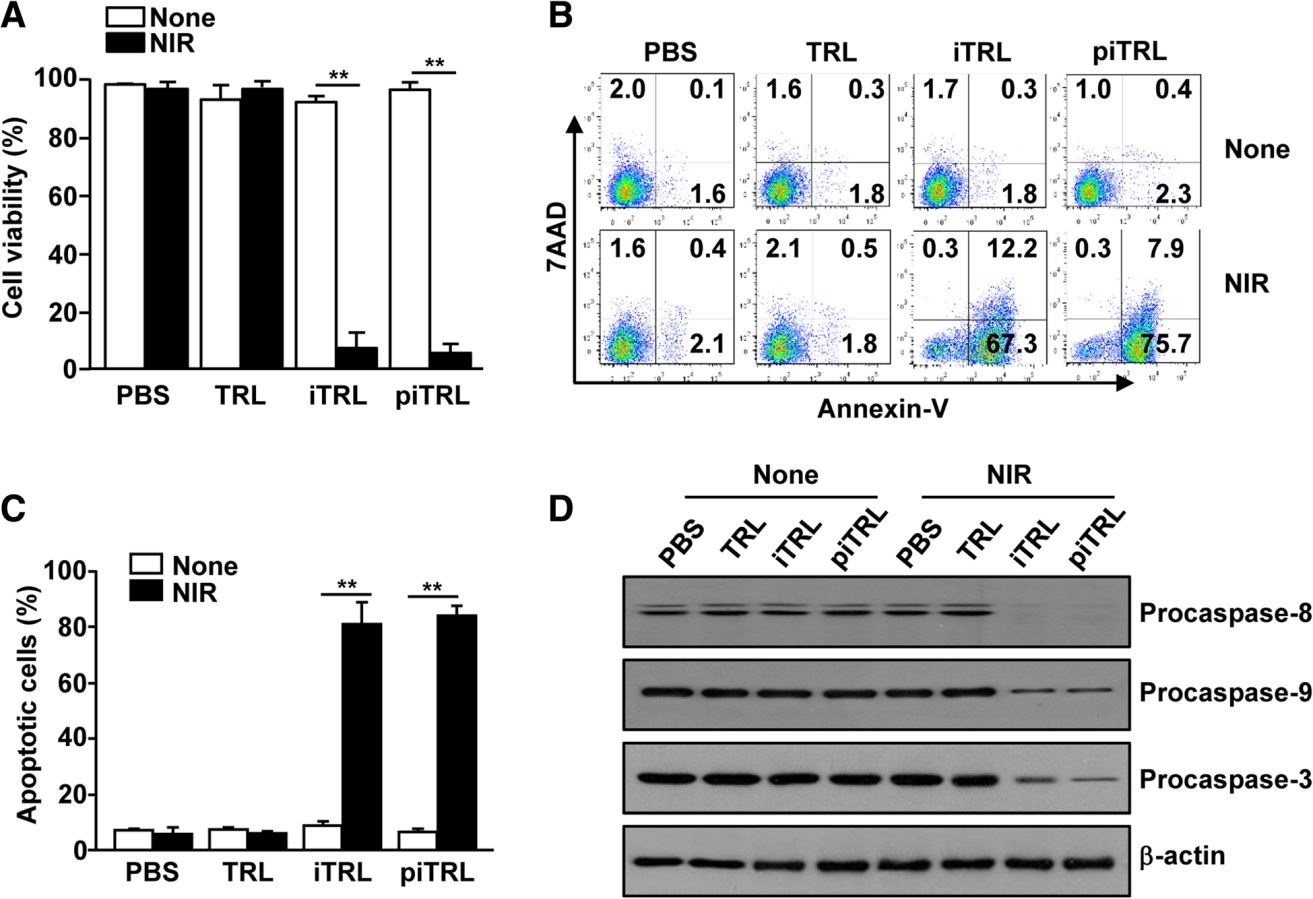
piTRL and laser irradiation promoted apoptosis of CT-26 carcinoma. CT-26 cells were incubated with PBS, TRL, iTRL or piTRL for 1 h, and the cells were treated with or without laser irradiation at 1 W/cm^2^ for 5 min and cultured for 24 h. **a** Cell viability of CT-26 was measured by MTT assay; ** p < 0.01. **b** Apoptosis was analyzed by annexin-V and 7AAD staining on flow cytometry. **c** Mean percentages of apoptotic cells, ** p < 0.01. **d** The expression levels of procaspase-8, − 9, and − 3 were assayed by western blotting analysis. β-actin was used as a loading control

Because laser irradiation increases the temperature of piTRLs, we next examined PTT efficiency against B16 melanoma and CT-26 carcinoma cells. Before evaluation of the photothermal effect, we studied the toxicity of the liposomes in the Raw 264.7 cells and found that the treatment with liposomes did not have any toxic effect on the cells (Additional file 1: Figure S1). Moreover, treatment of CT-26 cells with liposomes did not induce cell death in the absence of laser irradiation whereas the viability of CT-26 cells was significantly decreased following laser irradiation of iTRL- and piTRL-treated cells (Fig. 2a). Laser irradiation and treatment with iTRLs and piTRLs induced apoptotic cell death as indicated by the increases in the number of annexin-V-positive and 7AAD-positive cells, whereas laser irradiation and TRL treatment did not induce apoptosis of CT-26 cells (Fig. 2b and c). In addition, treatment with iTRLs and piTRLs and laser irradiation resulted in the activation of apoptotic signaling pathway, since the levels of procaspase-3, − 8, and − 9, were dramatically decreased (Fig. 2d). Therefore, these results indicated that piTRL and iTRL can be used as photothermal molecules.

### piTRL and laser irradiation eliminated melanoma and carcinoma by photothermal therapy (PTT)

**Fig. 3:**
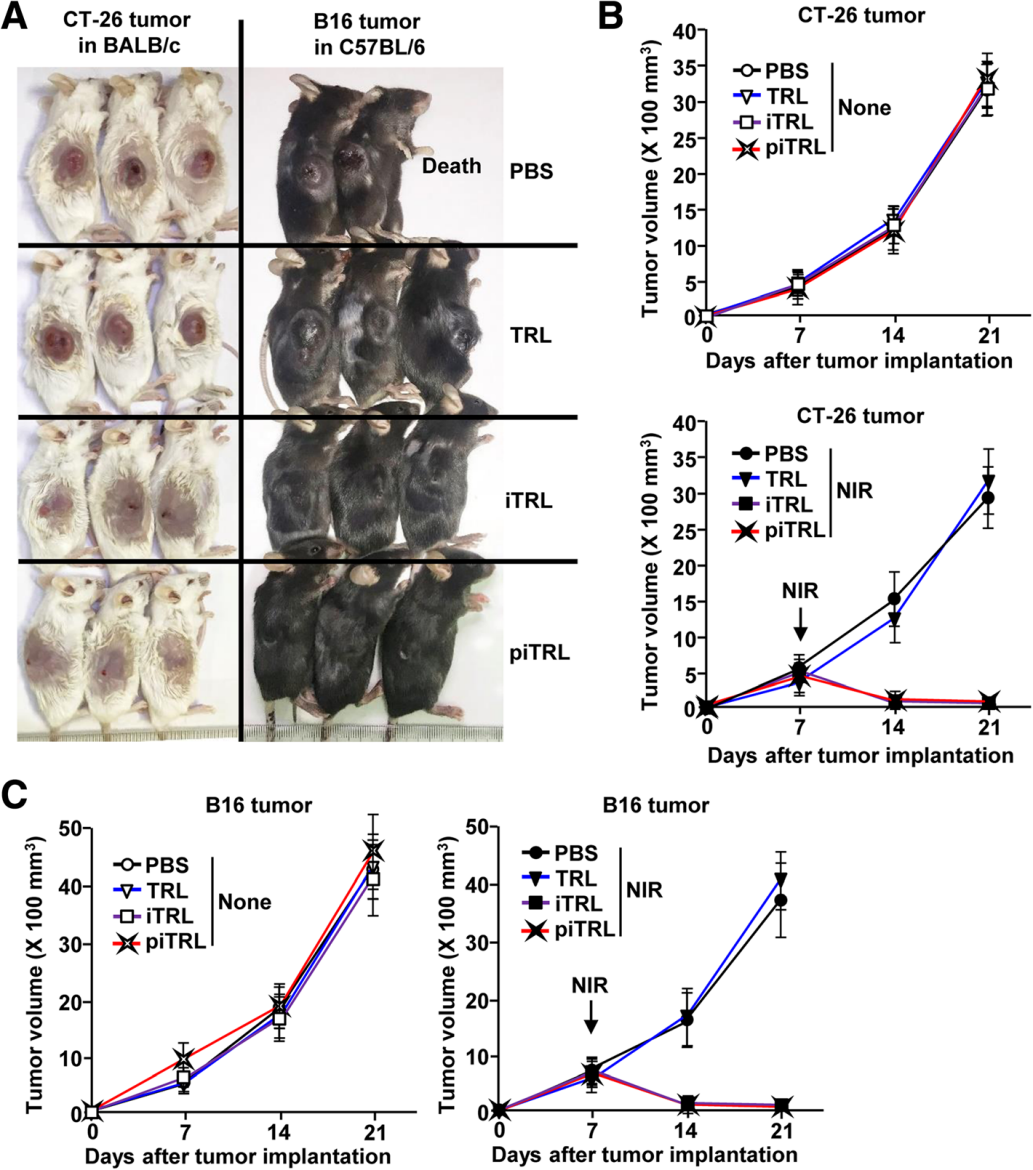
Anti-cancer effect of piTRL treatment followed by laser irradiation against CT-26 carcinoma and B16 melanoma. BALB/c and C57BL/6 mice were subcutaneously (*s.c.*) inoculated with 1 × 10^6^ CT-26 and B16 cells, respectively. The mice were intratumorally (*i.t.*) injected with PBS, TRL, iTRL, or piTRL on day 7 of tumor cell injection and were treated with or without laser irradiation at a power density of 1 W/cm^2^ for 5 min. **a** CT-26 (left panel) and B16 (right panel) tumor mass is shown on day 21 of tumor injection. **b** Tumor growth curves for CT-26 carcinoma with or without laser irradiation. **c** B16 melanoma tumor growth curves for the mice in the presence or absence of laser irradiation. Data are from the analyses of six individual mice (three mice per experiment for a total of two independent experiments)

Since the liposomes could induce cancer cell apoptosis, we next examined whether they can be used in the treatment of tumors in mice. For the evaluation of the anti-tumor effect of the liposomes, we assessed CT-26 carcinoma in BALB/c mice and B16 melanoma in C57BL/6 mice. Once the tumors were established on day 7 after initial implantation of tumors, we *i.t.* administrated the liposomes in the mice and irradiated them with an 808 nm laser at 1 W/cm^2^ for 5 min. Laser irradiation of the iTRL- and piTRL-treated tumors increased their temperatures to 52.5 ± 1.4 °C and 53.1 °C ± 1.8 °C, respectively, whereas it did not change the temperatures of PBS- and TRL-treated tumors (Additional file 1: Figure S2). Moreover, the tumors in BALB/c and C57BL/6 almost disappeared on day 21 after tumor injection by treatment with iTRLs and piTRLs and laser irradiation (Fig. 3a). In addition, the tumor growth also efficiently inhibited in BALB/c and C57BL/6 mice after treatment with iTRL and piTRL and laser irradiation (Fig. 3b and c). Thus, these data suggested that laser irradiation of iTRL- or piTRL-treated tumors promotes PTT-mediated anti-cancer effects in cases of carcinoma and melanoma.

### piTRL treatment with laser irradiation promoted dendritic cell (DC) activation in the tumor draining lymph node (tdLN)

**Fig. 4:**
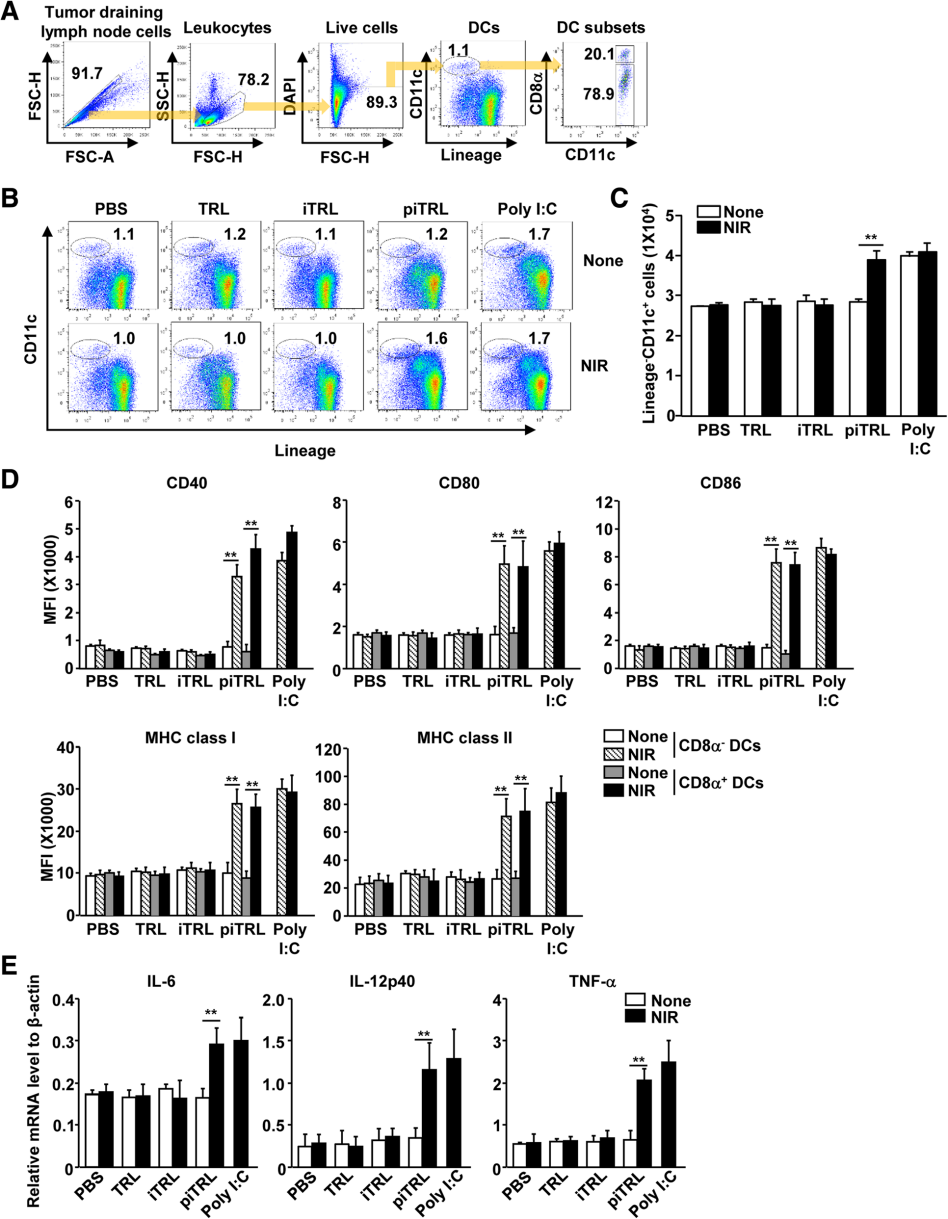
piTRL treatment followed by laser irradiation promoted activation in tumor-draining lymph node (tdLN). CT-26 tumor-bearing mice were *i.t.* injected with PBS, TRL, iTRL, piTRL or poly I:C, and treated with or without laser irradiation for 5 min. tdLN were harvested 24 h after laser irradiation. **a** Definition of DC population in tdLN was shown. Lineage markers included CD3, Thy1.1, B220, Gr-1, CD49b, and TER-119. Lineage^−^CD11c^+^ DCs were further divided as CD8α^+^ and CD8α^−^ DCs. **b** Frequency of tdLN DCs is shown. **c** Mean absolute number of Lineage^−^CD11c^+^ cells in tdLN is shown, ** p < 0.01. **d** Mean fluorescence intensity (MFI) of co-stimulatory molecules and MHC class I and II in gated CD8α^+^ and CD8α^−^ DCs in tdLN was analyzed using flow cytometry. **e** Levels of IL-6, IL-12p40 and TNF-α mRNA in tdLN. All data are representative of the average of the analyses of six independent samples (i.e., three samples per experiment, two independent experiments)

Next, we evaluated the effect of piTRL-released poly I:C on lymph node (LN) dendritic cell (DC) activation. We *i.t.* injected liposomes in the CT-26 tumor-bearing BALB/c mice and irradiated them with an 808 nm laser at 1 W/cm^2^ for 5 min. Twenty-four hours after laser irradiation, tumor-draining LNs (tdLN) were harvested and analyzed for DC activation. The tdLN DCs were defined as lineage^−^CD11c^+^ cells in live leukocytes, and the DCs were further divided into CD8α^+^ and CD8α^−^ DCs from the lineage^−^CD11c^+^ cells (Fig. 4a). The mice treated with PBS, TRL, or iTRL in the presence or absence of laser irradiation did not show a change in the population and number of tdLN DCs. In contrast, treatment with piTRLs and laser irradiation promoted substantial increases in the frequency and number of DCs in tdLNs, the levels of which were almost similar to those obtained following treatment of mice with 20 μg of poly I:C (Fig. 4b and c). In addition, piTRL treatment and laser irradiation induced dramatic increases in the levels of co-stimulatory molecules and the expression of major histocompatibility complex (MHC) class I and II in tdNL CD8α^+^ and CD8α^−^ DCs (Fig. 4d). Furthermore, the mRNA levels of pro-inflammatory cytokines, interleukin-6 (IL-6), IL-12p40 and tumor necrosis factor-α (TNF-α) in the tdNL were also significantly increased by treatment with piTRL and laser irradiation compared to those in controls (Fig. 4e). In addition, the mRNA levels of interferon-γ (IFN-γ) and T-bet, a transcription factor of Th1 cells, were also up-regulated by the piTRL treatment and laser irradiation, whereas the levels of Th2- and Th17-associated mRNA, GATA3, and RORγt were not changed (Additional file 1: Figure S3). Thus, these results suggested that poly I:C released from piTRLs upon laser irradiation induced activation of DCs in the mice in vivo.

### Laser irradiation in piTRL-treated mice prevented metastatic cancer in lung

**Fig. 5:**
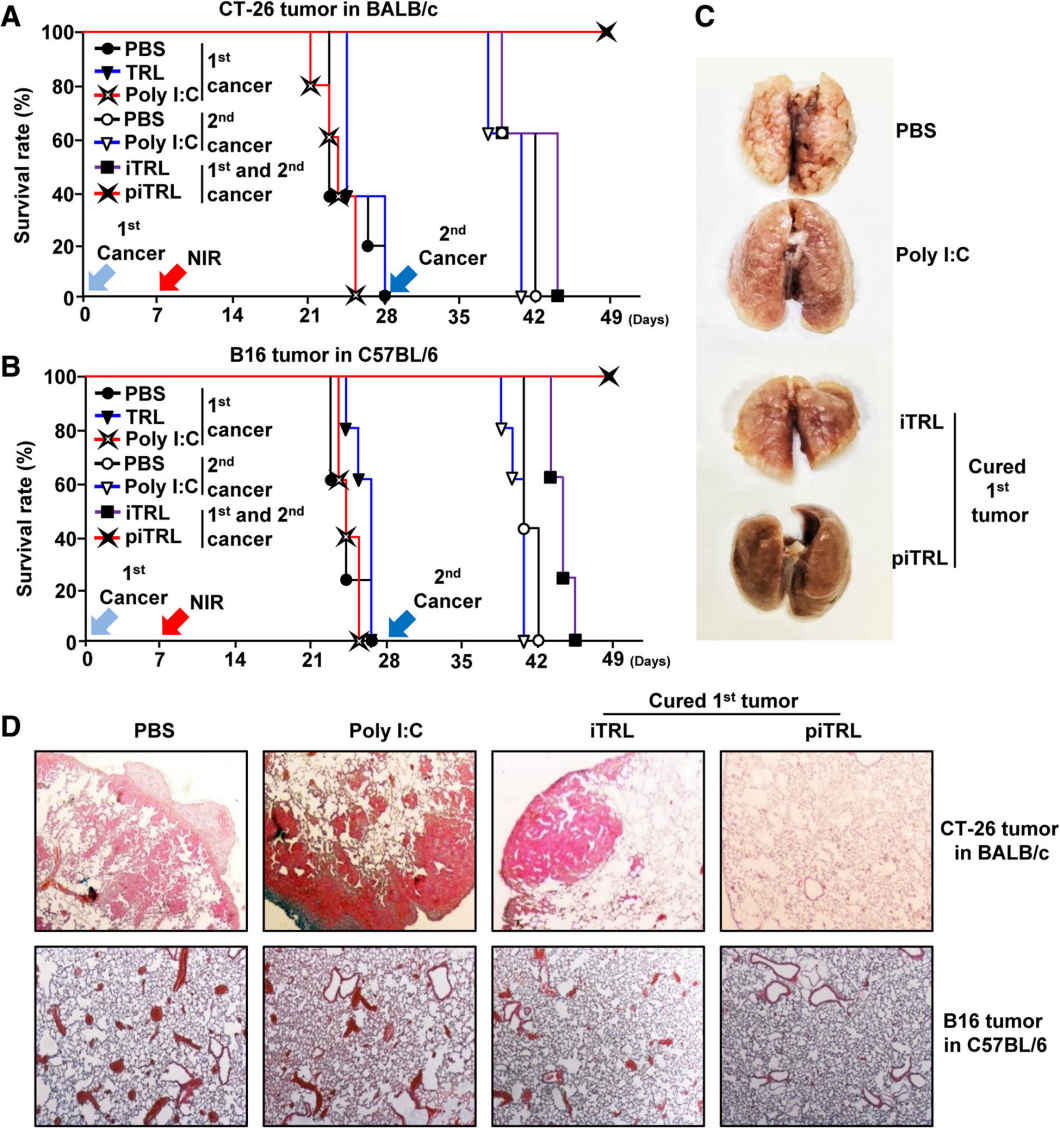
Protective effect of piTRL treatment with laser irradiation against lung metastasis of cancer. On day 28 of the 1st transplanted tumor challenge, mice treated with iTRL or piTRL andlaser irradiation mice were further intravenously (*i.v.*) inoculated 2nd transplant of CT-26 and B16 cells, respectively. PBS- and poly I:C-treated mice were also *i.v.* injected with the cancer cells as a control. **a** The survival rate of CT-26 challenged BALB/c mice and **b** B16 challenged C57BL/6 mice were monitored, *n = 5* for each group. **c** Representative images of CT-26 metastatic lung cancer. **d** H&E staining of lung on day 10 of 2nd transplant of CT-26 and B16 cell challenge. Data are representative of the analyses of six independent samples (i.e., three mice per experiment, two independent experiments)

Our data showing that piTRL treatment with laser irradiation was effective therapy against the growth of the 1st transplanted tumor and induced DC activation in tdLN motivated us to examine the anti-metastatic effect of piTRLs. On day 28 of 1st tumor injection, the BALB/c and C57BL/6 mice were cured by iTRL and piTRL treatment and laser irradiation and *i.v.* injected with 0.5 × 10^6^ CT-26 or B16 cells for establishing metastatic models as 2nd transplantation of tumors, respectively. PBS and TRL treatment with laser irradiation did not inhibit the growth of the 1st transplanted tumors, and these mice consequently dyed within 28 days of 1st tumor transplantation (Fig. 5a, b). The mice cured of the 1st transplanted tumors by piTRL treatment with laser irradiation survived from the 2nd *i.v.* tumor cell challenge during monitoring, whereas the mice cured by iTRL-mediated treatment died within 18 days of 2nd injection of cancer cells (Fig. 5a, b and Additional file 1: Figure S4). Moreover, cancer cell infiltration in the lung was substantially inhibited in mice treated with piTRL and laser irradiation compared to control mice treated with PBS, poly I:C, or iTRLs (Fig. 5c and d). The mice cured from the CT-26 tumor by treatment with piTRL-mediated PTT were not been protected against the 2nd challenge with 4 T1 breast cancer cells (Additional file 1: Figure S4B), indicating that the protective effect of piTRL against the 2nd transplanted cancer depends on the 1st transplanted tumor.

**Fig. 6:**
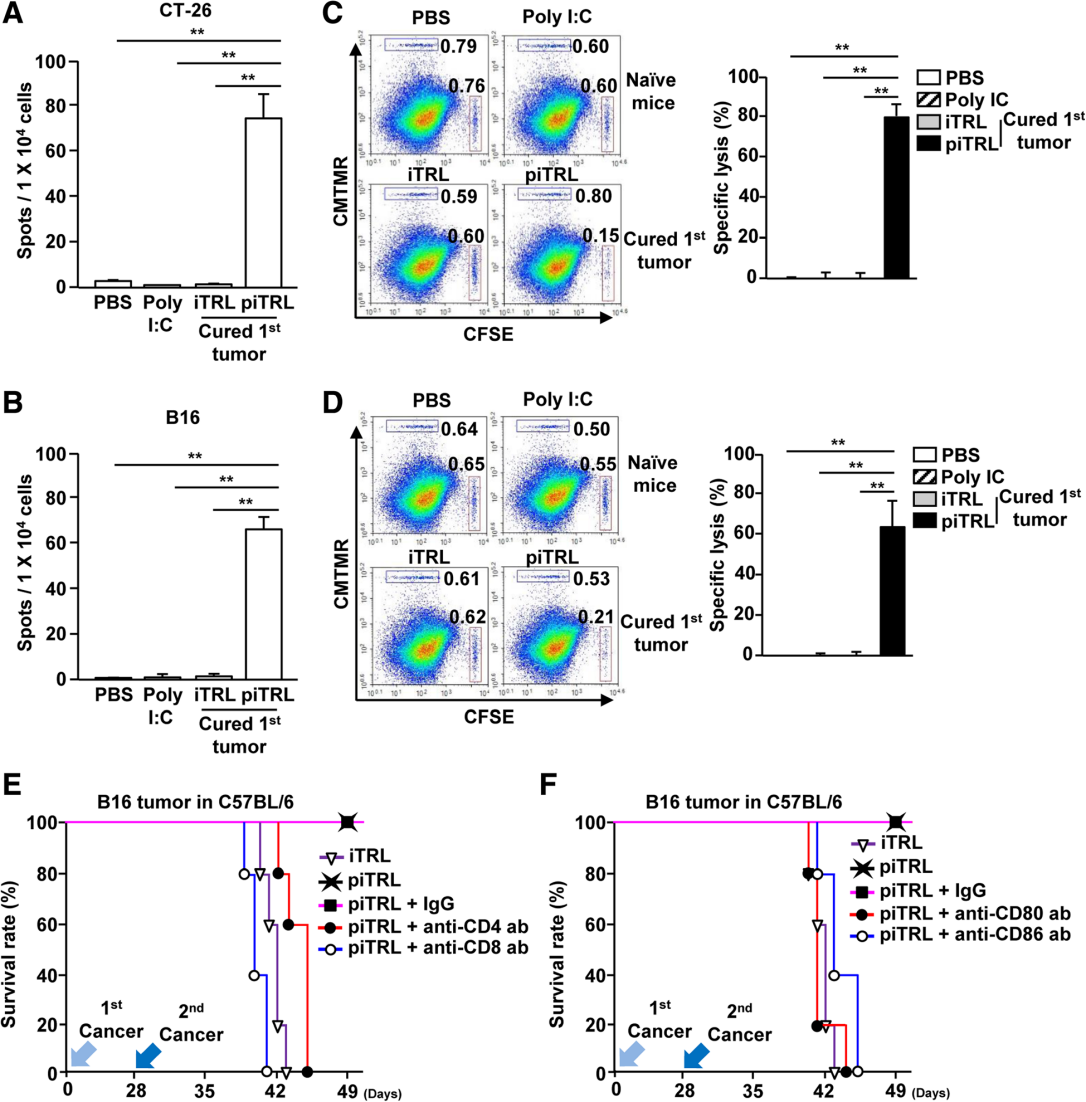
Induction of cancer Ag-specific immune responses by piTRL. BALB/c and C57BL/6 mice were subcutaneously injected with cancer cells (1st transplanted tumor) and treated by the liposomes as shown in Fig. 5. **a** and **b** Spleens were harvested on day 10 of the 2nd transplantation of tumor. The splenocytes were stimulated with **a** CT-26 or **b** B16 self-Ag for 24 h and IFN-γ production was measured by ELISPOT. ** p < 0.01. **c** and **d** Specific lysis of cells was analyzed on day 10 of the 2nd transplantation of tumor in mice by transferring cancer Ag- or control peptide-coated splenocytes. ** p < 0.01. **e** and **f** B16 tumors in C57BL/6 mice were treated with piTRL and laser irradiation as indicated in Fig. 5. On day 25 of the 1st transplant of B16 cells, the mice received **e** depletion abs (anti-CD4 and anti-CD8 abs) or **f** blockade abs (anti-CD80 and anti-CD86 abs). The curves show survival rates of mice (*n = 5* for each group)

Next, we evaluated whether the rejection of the 2nd transplanted cancer depended on self Ag-specific immune responses. On day 35 after1^st^ tumor injection, which was day 7 of the 2nd tumor administration, spleen was harvested and splenocytes were incubated with the self-Ag of CT-26 or B16, respectively, for 24 h. The mice treated with piTRL and laser irradiation showed significant increases in IFN-γ production in response to self-Ags, whereas other control treated mice did not show production of IFN-γ (Fig. 6a and b). The mice survived from the 1st transplanted tumor due to piTRL treatment showed significantly higher specific lysis of tumor Ag-coated splenocytes than iTRL and poly I:C treated mice whose splenocytes were not killed (Fig. 6c and d). Moreover, lung infiltrated T cells in piTRL-treated mice that were cured from the 1st transplanted tumor produced much higher amounts of IFN-γ and TNF-α in response to cancer Ag (Additional file 1: Figure S5). In addition, depletion of CD4 and CD8 T cells by Ab treatment in piTRL-treated mice that were cured from the 1st transplanted tumor failed to protect from the 2nd transplanted cancer (Fig. 6e). Blockade of co-stimulatory molecules in mice cured from 1st transplanted tumor by piTRL also failed to protect against the 2nd transplanted cancer (Fig. 6f). Thus, these results demonstrate that the protective effect of piTRL against the 2nd transplanted cancer depends on cancer Ag-specific immune responses. Immune cell infiltration in the peripheral tissues such as the colon, kidneys, and liver was not detected in cases of piTRL treatment with laser irradiation, which indicated that piTRL with laser irradiation did not promote inflammation in peripheral tissue (Additional file 1: Figure S6). Therefore, these data suggested that the treatment of piTRL with laser irradiation cured both the 1st transplanted cancer and the 2nd transplanted cancer.

## Discussion

Since liposomes have low cytotoxicity in both animals and humans, they have been extensively studied as delivery vehicles for anti-cancer drugs. The sensitivity of liposomes to temperature is an especially attractive feature since they can release encapsulated molecules in the space around the physiological temperature. Upon NIR laser-mediated increase in temperature to 42 °C, the membrane of TRLs becomes permeable, such that the encapsulated molecules are released [24, 28]. TRLs have been used with PTT and anti-cancer drug-induced chemotherapy for cancer [24]. In this study, we used a TRL system in which ICG was incorporated in the bilayer and poly I:C was encapsulated. The ICG efficiently reacted to NIR laser irradiation by increasing the temperature and effectively releasing poly I:C. Therefore, the piTRLs may be used for PTT and immunotherapy against cancer and its metastasis.

Immunotherapy aims to promote Ag-specific immune responses against cancer Ags that lead to efficient and selective killing of cancer cells [29, 30]. Ag-specific immune responses are controlled by Ag presenting cells such as DCs, macrophages, and B cells [29, 30]. Among these, DCs are the most potent Ag presenting cells [31]. In mice, myeloid types of DCs contained two main subsets: CD8α^+^ and CD8α^−^ DCs. The CD8α^+^ DCs are specialized in the cross-presentation of Ag to CD8^+^ T cells, which are primed for cytotoxic T lymphocyte (CTL) response. On the other hand, CD8α^−^ DCs present exogenous Ag to CD4^+^ T cells, then developing into helper T (Th) cells for the production of cytokines [32–34]. These subsets of DC activation are essential for Ag-specific immunotherapy against cancer. We found that piTRL treatment with laser irradiation induced both CD8α^+^ and CD8α^−^ DC activation. Taken together with the PTT-induced apoptosis of tumor cells, the stimulatory effect of piTRLs in tdLN DCs may promote Ag-specific immune responses for protection against cancer metastasis.

It has been found that PTT induces cancer cell apoptosis [6, 35]. Apoptosis is programmed cell death, and cancer Ags are generated by the apoptosis of cancer cells [7]. Although many studies have attempted to induce the apoptosis of cancer cells, the cancer cell apoptosis-generated molecules do not completely prevent metastasis because the cancer Ags are poorly immunogenic [23, 36]. While the iTRL treatment with laser irradiation successfully cured the1^st^ transplanted tumors in our study, it could not inhibit growth of the 2nd transplanted cancer growth in BALB/c and C57BL/6 mice. This failure of iTRL to provide protection against the2nd transplanted cancer may be due to less immune activation by apoptosis-generated molecules [36–39], as we have shown that iTRL treatment with laser irradiation did not promote DC activation in tdLNs and specific killing of cancer Ag-coated splenocytes. In contrast, piTRL-designed to release poly I:C upon laser irradiation induced activation of tdLN DCs. Moreover, PTT-induced apoptosis of tumor cells produces tumor Ags, and the released poly I:C may promote tumor Ag-specific immune activation. This consequently may have prevented growth of the 2^nd^ transplanted cancer in mice cured of the 1st transplanted tumors. Moreover, depletion of T cells and blockade of co-stimulatory molecules failed to protect mice from the 2nd transplanted cancer. Taken together, these results demonstrated that the piTRL-induced protective effect against the 2nd transplanted cancer was mediated by DC and T cell activation. We also found that within 24 h, 40% of the encapsulated poly I:C was released from piTRL without laser irradiation at 30 °C; however, it did not induce activation of DCs in the tdLN. This may be due to two reasons. First, immune stimulatory amount of poly I:C is 20 μg in the mouse in vivo, but the amount of the poly I:C spontaneously released from the liposomes was 8 μg, which may not be enough to induce DC activation. Second, the spontaneous release of poly I:C may be very slow, which may promote immune tolerance against poly I:C. To evaluate the effect of the slow release of poly I:C on DC activation, we plan to synthesize poly I:C-containing hydrogel and examine the effect of DC activation in the mice in vivo.

## Conclusions

**Fig. 7:**
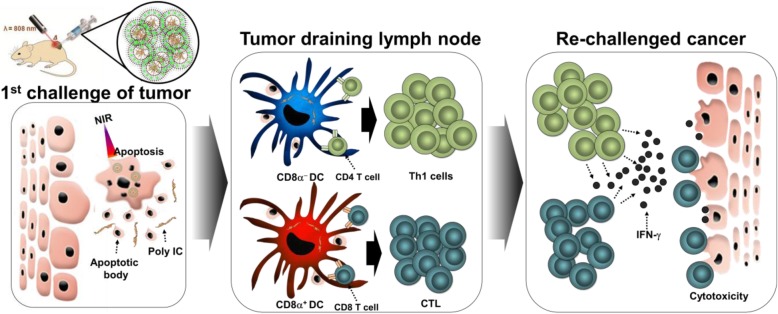
Schematic illustration of poly I:C and ICG containing temperature-sensitive liposome (piTRL) induced immuno-photothermal therapy for treatment of 1st and 2nd transplanted cancers

To develop novel therapeutic materials for combination therapy against cancer, nanoscience can be of great help [40, 41]. Since nanocarriers can carry appropriate molecules, it would be beneficial to combine new therapeutic molecule for delivery of those molecules to tumor for more complex trials [40–44]. As shown in Fig. 7, we developed a TRL containing a photothermal reagent, ICG, and an immune stimulatory molecule, poly I:C, which suitably increased temperatures against NIR laser irradiation for inducing PTT and releasing poly I:C for the promotion of immune activation. This combination eventually showed some success against subcutaneous tumors in a mouse model and against metastatic growth of carcinoma and melanoma in BALB/c and C57BL/6 mice via cancer Ag-specific immune activation. Thus, piTRL is a promising candidate for the treatment of cancer and prevention of metastasis as a photothermal and immunotherapy material.

## Additional file

Additional file 1: **Figure S1.** Cell viability upon liposome treatment. **Figure S2.** Changes in temperature in liposome-treated tumor by NIR laser irradiation. **Figure S3.** piTRL treatment and laser irradiation promoted IFN-γ production. **Figure S4.** Survival rate of CT-26 and B16-challenged BALB/c and C57BL/6 mice. **Figure S5.** Cancer antigen-specific IFN-γ and TNF-α production. **Figure S6.** Hematoxylin and eosin (H&E) staining of peripheral tissues. (DOCX 771 kb)

## Data Availability

The data supporting the conclusions of this article are displayed within the article and additional files. The actual raw data are available from the corresponding author on reasonable request.

## Abbreviations

Ag: Antigen
CTL: Cytotoxic T lymphocyte
DC: Dendritic cell
ED: Electron diffraction
FE-TEM: Field emission transmission electron microscopy
ICG: Indocyanine green
IFN: Interferon
IL: Interleukin
LN: Lymph node
MHC: Major histocompatibility complex
NIR: Near-infrared
Poly I:C: Polyinosinic:polycytidylic acid
PTT: Photothermal therapy
Th: Helper T
TNF: Tumor necrosis factor
TRL: Thermo-responsive liposome
