# Supplementary material for: Indocyanine green and poly I:C containing thermo-responsive liposomes used in immune-photothermal therapy prevent cancer growth and metastasis
Source: J Immunother Cancer. 2019 Aug 14;7:220. doi: 10.1186/s40425-019-0702-1 (PMC6694491; doi:10.1186/s40425-019-0702-1)
Supplement: Supplementary file 1 — Figure S1. Cell viability upon liposome treatment. Figure S2. Changes in temperature in liposome-treated tumor by NIR laser irradiation. Figure S3. piTRL treatment and laser irradiation promoted IFN-γ production. Figure S4. Survival rate of CT-26 and B16-challenged BALB/c and C57BL/6 mice. Figure S5. Cancer antigen-specific IFN-γ and TNF-α production. Figure S6. Hematoxylin and eosin (H&E) staining of peripheral tissues. (DOCX 771 kb) [file 40425_2019_702_MOESM1_ESM.docx]

Indocyanine green and poly I:C-containing thermo-responsive liposomes used in immuno-photothermal therapy prevent cancer growth and metastasis

Li Xu^1#^, Wei Zhang^1#^, Hae-Bin Park^2#^, Minseok Kwak^3^, Junghwan Oh^4,5,6^, Peter C.W. Lee^7^, and Jun-O Jin^1,2*^


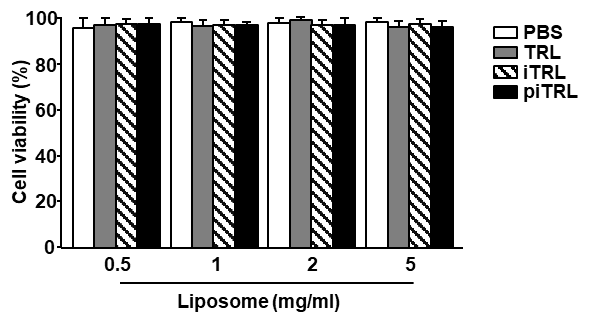


Figure S1. Cell viability upon liposome treatment. Raw cells (2 × 10^4^) were incubated with PBS, TRL, iTRL, or piTRL for 24 h and viability of cells was measured by MTT assay. Data are the average of six independent samples.


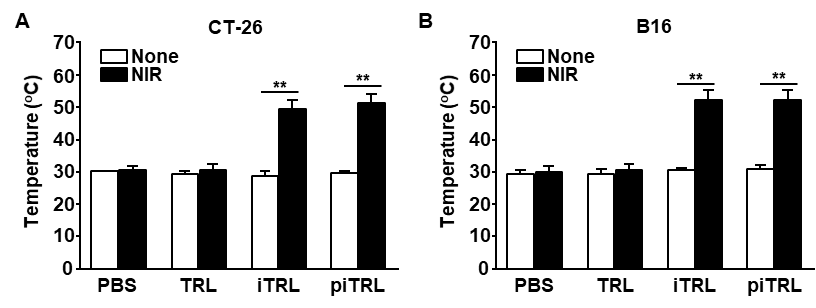


Figure S2. Changes in temperature in liposome-treated tumor by NIR laser irradiation. CT-26 and B16 tumor-bearing BALB/c and C57BL/6 mice were treated with PBS, TRL, iTRL, or piTRL for 1 h, and irradiated with NIR laser at 1 W/cm^2^ for 5 min. (A) Temperatures were measured after laser irradiation in CT-26 and (B) B16 tumor. Data are the average of six independent samples.


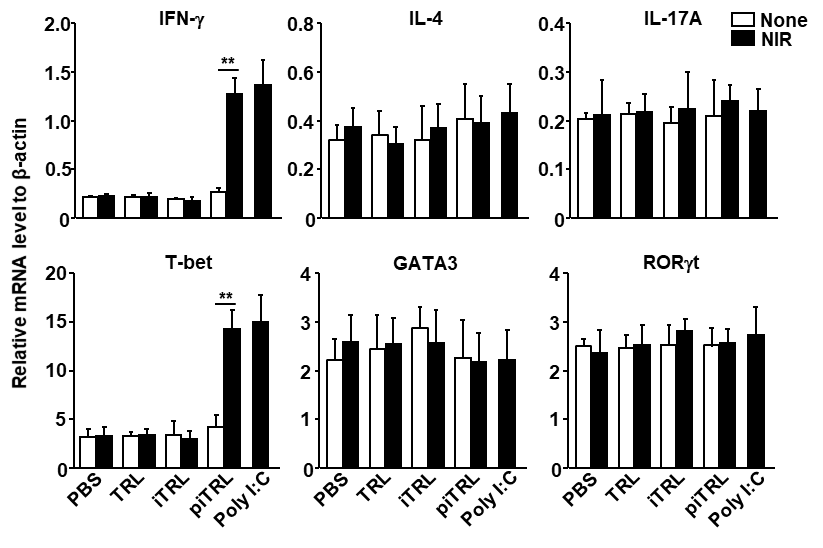


Figure S3. piTRL treatment and laser irradiation promoted IFN-γ production. BALB/c mice were treated with liposomes as indicated in Figure 3. The levels of the indicated mRNAs in tdLN were measured, *** p* < 0.01. Data are the average of six independent samples.


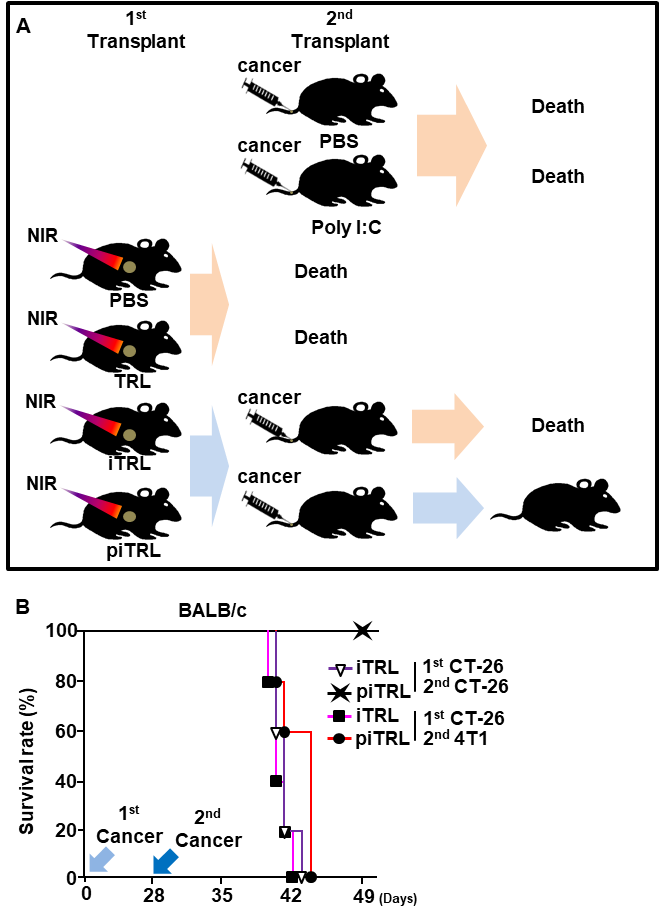


Figure S4. Survival rate of CT-26 and B16-challenged BALB/c and C57BL/6 mice. (A) On day 28 of 1^st^ tumor injection. Cured mice were challenge with same cancer cells by intravenous injections. Schematic diagram of 1^st^ and 2^nd^ tumor challenge and treatment. (B) BALB/c mice that had developed the CT-26 tumor were treated with iTRL or piTRL by PTT. The mice were 2^nd^ transplanted CT-26 or 4T1 cells. The survival rates are shown. *n=4* for each group.


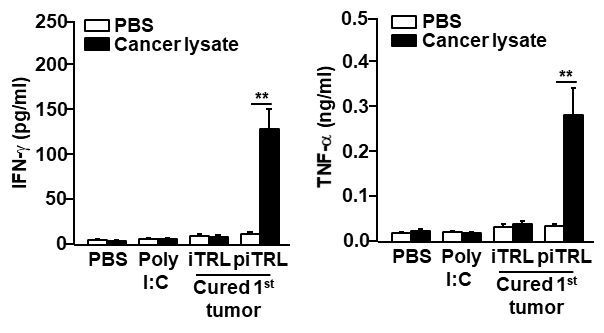


Figure S5. Cancer antigen-specific IFN-γ and TNF-α production. C57BL/6 mice were cured from the B16 tumor by piTRL and laser irradiation. On day 38 of 1^st^ tumor challenge, lung was harvested and cultured with lysates of B16 cells. Cytokine levels were measured in the cultured medium by ELISA.


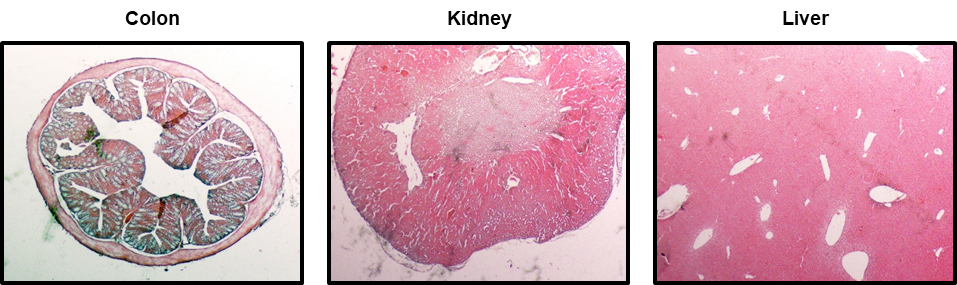


Figure S6. Hematoxylin and eosin (H&E) staining of peripheral tissues. BALB/c mice were inoculated twice with CT-26 cells as shown in Figure 5A. On day 38 of 1^st^ tumor injection, piTRL-treated plus laser-irradiated mice were sacrificed and colon, kidney and liver were harvested and the tissues stained with H&E.
